# Loss of Tyrosine Phosphatase Mu Promotes Scoliosis Progression Through Osteopontin-α5β1 Integrin Signaling and PIPK1γ90 Activity

**DOI:** 10.3390/ijms26031042

**Published:** 2025-01-26

**Authors:** Mohamed Elbakry, Nasrin Khatami, Marie-Yvonne Akoume, Cédric Julien, Saadallah Bouhanik, Anita Franco, Iurie Caraus, Wesam Elremaly, Alain Moreau

**Affiliations:** 1 Viscogliosi Laboratory in Molecular Genetics of Musculoskeletal Diseases, Azrieli Research Center, CHU Sainte-Justine, Montréal, QC H3T 1C5, Canada; 2 Department of Chemistry, Biochemistry Section, Faculty of Science, Tanta University, Tanta 31527, Egypt; 3 Department of Biochemistry and Molecular Medicine, Faculty of Medicine, Université de Montréal, Montréal, QC H3T 1J4, Canada; 4 Department of Cellular, Molecular Biology and Genetics, Faculty of Medicine, Université des Sciences de la Santé (USS) de Libreville, Libreville BP 18231, Gabon; 5 Department of Stomatology, Faculty of Dentistry, Université de Montréal, Montréal, QC H3T 1J4, Canada

**Keywords:** adolescent idiopathic scoliosis, osteoblast, PTPµ, PIPK1γ90, OPN-α5β1 integrin, Gi-coupled receptor signaling, spinal deformity, PTPµ-null mice, *PTPRM* variants, microRNAs

## Abstract

Adolescent idiopathic scoliosis (AIS) is characterized by a curvature of the spine affecting approximately 4% of the pediatric population, and the mechanisms driving its progression remain poorly understood. Whole-exome sequencing of a French-Canadian AIS cohort with severe scoliosis identified rare variants in the *PTPRM* gene, which encodes Protein Tyrosine Phosphatase μ (PTPµ). However, these rare variants alone did not account for the pronounced reduction in PTPµ at both mRNA and protein levels in severe AIS cases. This led us to investigate epigenetic regulators and the identification of five microRNAs (miR-103a-3p, miR-107, miR-148a-3p, miR-148b-3p, and miR-152-3p) that target *PTPRM* mRNA. These microRNAs were significantly elevated in plasma from severe AIS patients, and miR-148b-3p was also upregulated in AIS osteoblasts. Phenotypic analysis of bipedal *Ptrprm* knockout (PTPµ −/−) mice showed increased prevalence and severity of scoliosis, while quadrupedal PTPµ −/− mice did not develop scoliosis, underscoring PTPµ’s role as a disease-modifying factor. Mechanistically, PTPµ deficiency was found to disrupt Gi-coupled receptor signaling in osteoblasts by enhancing the interaction between osteopontin (OPN) and α5β1 integrin, along with increased tyrosine phosphorylation of phosphatidylinositol-4-phosphate 5-kinase type I (PIPKIγ90). These findings provide novel insights into the molecular mechanisms underlying spinal deformity progression in AIS, linking PTPµ depletion to aberrant OPN-α5β1 integrin signaling and highlighting potential therapeutic targets to stop, mitigate, or prevent scoliosis.

## 1. Introduction

Adolescent idiopathic scoliosis (AIS) is a complex, three-dimensional spinal deformity characterized by lateral curvature and vertebral rotation [1]. Affecting 0.47–5.2% of the global pediatric population, AIS is one of the most common childhood deformities [1]. AIS typically emerges during the early stages of puberty, with a prevalence of 1% to 4% in this age group, and exhibits a higher incidence in females compared to males [1]. Despite substantial research progress, the exact etiology and pathogenesis of AIS remain elusive [2,3,4,5]. It is widely accepted that the condition arises from multifactorial influences, including genetic [5], epigenetic [6], and mechanotransduction impairments [7,8,9].

Recent studies have demonstrated a systemic dysfunction in G inhibitory (Gi)-coupled receptor signaling in AIS pathogenesis [10,11,12,13,14], offering a potential avenue to advance the understanding of disease-modifying factors driving the progression of spinal deformities in AIS. By identifying and targeting specific genetic and molecular factors involved in the development and worsening of scoliosis, *PTPRM* gene emerged as a promising candidate. The *PTPRM* gene encodes the protein tyrosine phosphatase receptor type M (PTPµ). PTPµ is a member of the receptor-type protein tyrosine phosphatase family, which plays a crucial role in regulating cell–cell adhesion, migration, and signaling [15]. It is expressed in various tissues, including the nervous system and bone. PTPµ is involved in the dephosphorylation of tyrosine residues on proteins, thereby modulating signaling pathways related to cell growth, differentiation, and adhesion. Its function is significant in maintaining cellular homeostasis and tissue architecture, and alterations in PTPµ activity have been linked to various diseases, including cancer and skeletal disorders, which some may also manifest a scoliosis phenotype [16,17]. This study highlights the regulatory role of PTPµ on Gi-coupled receptor signaling inhibition induced by OPN-α5β1 interaction and the mechanism underlying spinal deformity progression in AIS.

## 2. Results

### 2.1. Clinical and Demographic Characteristic of Participants

This is a prospective cross-sectional study conducted in three pediatric spine centers. The participants were recruited from June 2002 to August 2013, and data collection was completed in June 2016. Non-scoliotic healthy subjects were recruited during the same period from primary and high schools from the Greater Montreal’s area. A summary of clinical and demographic features of our French-Canadian cohorts is provided in Table 1. As expected, there were more females in AIS patients than in healthy controls (Fisher’s exact test *p* = 0.001). Stratification by scoliosis severity was determined only in the participants who had reached their skeletal maturity, which resulted in 111 AIS patients (94F/17M) as severe cases (Cobb angle ≥ 45°), 117 AIS patients (120F/27M) as non-severe cases (Cobb angle 10°–44°), and 143 matched healthy controls (67F/76M). As detailed in Table 1, despite attempting to match controls to AIS patients based on age and sex, statistically significant differences were observed. Both progressor and non-progressor AIS patients were significantly older than the healthy controls (*p* < 0.001 for both comparisons). This age difference was also evident within each sex: female progressor AIS patients (*p* < 0.001), female non-progressor AIS patients (*p* < 0.001), male patients progressor AIS patients (*p* < 0.001), and male non-progressor AIS patients (*p* < 0.001) were all older than their respective control groups. These findings highlight the challenges in achieving perfect age and sex matching in AIS studies, especially given the condition’s known predilection for females.

For miRNA analysis, plasma samples were collected at the first medical visit from symptomatic AIS patients and based on their clinical outcomes at skeletal maturity, these patients were categorized as either severe/progressors (P) or non-severe/non-progressors (NP). At the first medical visit, the progressor group (P) included 36 AIS patients (27F/9M) with a mean Cobb angle of 32.6° ± 8.1°, while the non-progressor group (NP) included 28 AIS patients (16F/12M) with a mean Cobb angle of 18.6° ± 6.1°. Additionally, 28 healthy controls (15F/13M) without scoliosis were included in the study (Supplementary Table S1). For in vitro analyses, osteoblast samples were derived and obtained by intraoperative bone biopsies of 11 female AIS surgical cases with a mean Cobb angle of 59.7° ± 10.5° and three female non-scoliotic trauma cases as controls (Supplementary Table S2).

### 2.2. Search for Variants Associated with AIS and Scoliosis Severity

Whole-exome sequencing (WES) of a French-Canadian cohort, including 70 AIS females with severe scoliosis (surgical cases) and 69 age-matched healthy females as controls, revealed three rare variants (MAF < 0.01) within the *PTPRM* gene (Supplementary Table S3). Due to the very low frequency of these variants, we analyzed six additional single nucleotide polymorphisms (SNPs), rs35224276, rs1247471646, rs763119679, rs766403029, rs368817148, and rs12112955204, in 148 severe AIS cases (Cobb angle ≥ 45°), 117 mild-to-moderate AIS cases (10°–44°), and 137 healthy controls, following ancestral and relatedness testing. These six SNPs were selected because of their potential to affect the *PTPRM* gene’s expression, mRNA splicing or stability, and the functional integrity of the PTPµ protein. Unfortunately, none of these SNPs were found to be associated with scoliosis severity or AIS (Supplementary Table S4). Nevertheless, the discovery of the initial three rare variants associated with AIS and scoliosis severity prompted us to explore the expression and protein levels of PTPµ in osteoblasts derived from AIS patients (surgical cases).

### 2.3. Reduction in PTPµ Protein Occurs in Osteoblasts from Patients with AIS

**Figure 1:**
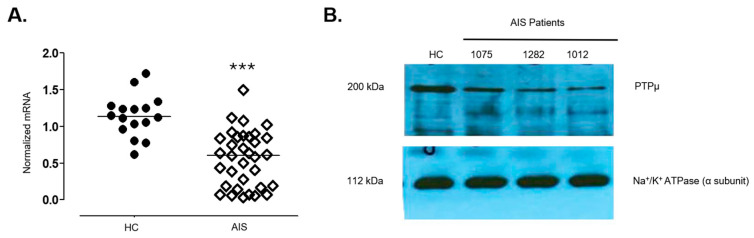
Analysis of *PTPRM* gene and protein expression in osteoblasts derived from scoliotic and non-scoliotic subjects. Panel (**A**) illustrates a qPCR analysis to assess the expression levels of *PTPRM* in primary osteoblasts derived from scoliotic patients (AIS) compared to non-scoliotic trauma cases considered here as healthy controls (HC) and revealed a two-fold reduction in *PTPRM* mRNA level in osteoblasts from scoliotic patients compared to the HC group (*** *p* > 0.001, *t*-test). Panel (**B**) shows a representative Western blot analysis of cell lysates demonstrating a 50% reduction in PTPµ protein levels in osteoblasts from idiopathic scoliosis patients compared to non-scoliotic controls. The experiment was repeated multiple times using different healthy controls and AIS patients. Another representative blot is shown in Supplementary Figure S1, showing 4 HC and 4 AIS patients. PTPµ protein levels were quantified by densitometry using ImageJ software and normalized to Na+/K+ ATPase. PTPµ protein levels were significantly reduced in AIS osteoblasts compared to HC. Data are representative of multiple independent experiments.

We observed a significant reduction in *PTPRM* gene expression in osteoblasts from scoliotic patients compared to non-scoliotic trauma cases, as determined by qPCR (Figure 1A). All scoliotic patients examined showed approximately a two-fold decrease in *PTPRM* mRNA levels in their osteoblasts relative to non-scoliotic control subjects (*p* = 0.001). Western blot analysis of cell lysates similarly demonstrated a reduction in PTPµ protein levels in AIS osteoblasts compared to non-scoliotic controls (Figure 1B). PTPµ protein levels were quantified by densitometry using ImageJ software (version 2.0.0) and normalized to Na+/K+ ATPase. PTPµ protein levels were significantly reduced in AIS osteoblasts compared to HC (*p* = 0.02 *). These findings clearly indicate that PTPµ is downregulated in severe AIS patients.

### 2.4. Investigating Epigenetic Mechanisms Targeting PTPRM in AIS Pathogenesis

We explored the potential role of epigenetic mechanisms in the reduction in PTPµ in AIS osteoblasts, particularly in the absence of a clear genetic association. Our focus shifted to small non-coding RNAs (microRNAs) that regulate *PTPRM* post-transcriptionally. Using Ingenuity Pathway Analysis (IPA) software (version 51963813), we initially identified 97 microRNAs (miRNAs) predicted to target *PTPRM* (Supplementary Table S5). Genome-wide expression analysis with Agilent miRNA array technology revealed that 11 of these miRNAs were upregulated in the plasma of AIS patients (Supplementary Table S6). Further analysis confirmed that five of these miRNAs, miR-103a-3p, miR-107, miR-148a-3p, miR-148b-3p, and miR-152-3p, were significantly upregulated in plasma samples from severe AIS cases (progressors) compared to non-severe cases (non-progressors) (Supplementary Figure S2). When examining osteoblasts from surgical AIS cases versus non-scoliotic trauma controls, only miR-148b-3p showed a significant upregulation in AIS patients (Supplementary Figure S3A,B). Moreover, a high-affinity binding site for miR-148b-3p was localized with the 3′UTR region of *PTPRM* gene (Supplementary Figure S3C). Receiver operating characteristic (ROC) curve analysis revealed that these five miRNAs effectively distinguished severe scoliosis or disease progressors (Cobb angle ≥ 45°) from less severe cases (Cobb angle 10°–44°), with an area under the curve (AUC) of 95%, an accuracy of 92.3%, a sensitivity of 90%, and a specificity of 100% (Supplementary Figure S4).

To better understand the role of these miRNAs in the pathogenesis of AIS, we conducted a systematic analysis of gene pathways and networks. The IPA gene pathway analysis revealed that most of these five miRNAs are predominantly involved in muscle and skeletal diseases (Supplementary Figure S5). By combining IPA with manual literature curation, we identified genes and molecules previously associated with AIS or scoliosis and established connections between these and the five miRNAs using the IPA Ingenuity Knowledge Base. We enhanced the figure by including AIS-associated miRNAs from the literature that share common targets with *PTPRM*-related miRNAs. Additionally, we explored potential drugs associated with the five *PTPRM*-targeting miRNAs. This approach enabled us to construct detailed networks linking each miRNA to its targets, including AIS-related genes that may play critical roles in the development of AIS. Overall, this hybrid analysis facilitated the creation of a more comprehensive network connecting these miRNAs to *PTPRM*, along with other genes, functions, and disease targets (Supplementary Figure S5).

### 2.5. Assessment of PTPµ Function in Scoliosis Onset and Disease Progression

To investigate the role of PTPµ in scoliosis onset and spinal deformity progression, first we conducted a phenotypic analysis of *Ptprm*-null mice. Quadrupedal male and female mice lacking of PTPµ displayed no signs of scoliosis or skeletal abnormalities, indicating that PTPµ deficiency alone is insufficient to trigger scoliosis. Next, we utilized a bipedal scoliosis mouse model [18,19] where scoliosis is induced by forelimb and tail amputations. This model is associated with elevated levels of circulating osteopontin (OPN) [20], a pro-inflammatory glycoprotein involved in wound healing [21]. We compared scoliosis development in *Ptprm*-null and wild-type (WT) mice following the amputation procedure.

**Figure 2:**
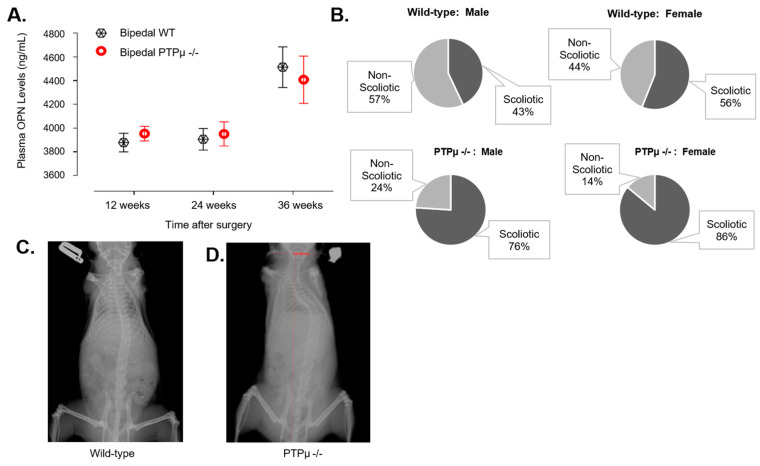
Effect of PTPµ-deficiency on plasma OPN and scoliosis in bipedal mice. Panel (**A**) represents a quantitative comparison of plasma osteopontin (OPN) levels in bipedal wild-type (WT) and PTPµ −/− mice. Plasma OPN levels were determined by ELISA at the indicated time point after bipedal surgery in 33 wild-type mice and 58 PTPµ −/− mice. Error bars represent standard deviation. Panel (**B**) summarizes the frequency of occurrence of scoliosis as induced by bipedal surgery in WT and PTPµ −/− mice. Panels (**C**,**D**) are representative spine radiographies showing a striking difference in spinal curvature between (**C**) bipedal WT and (**D**) bipedal PTPµ −/− mice.

We compared scoliosis development in *Ptprm*-null and wild-type (WT) mice following the amputation procedure. As expected, the amputation procedure significantly increased plasma OPN levels in scoliotic mice compared to non-scoliotic controls (Table 2). However, there was no difference in plasma OPN levels between *Ptprm*-null and WT mice that developed scoliosis (Figure 2A, Table 2).

Despite similar OPN levels, *Ptprm*-null mice exhibited a higher prevalence of scoliosis. At 36 weeks post-surgery, 81% of *Ptprm*-null mice developed scoliosis compared to 50% of WT mice (Figure 2B). Furthermore, *Ptprm*-null mice developed more severe scoliosis, as indicated by more pronounced lateral curvature (Figure 2C,D). These findings highlight the critical role of PTPµ in the progression of spinal deformities.

### 2.6. PTPµ Deficiency Worsens OPN-Mediated Gi Receptor Signaling Dysfunction

**Figure 3:**
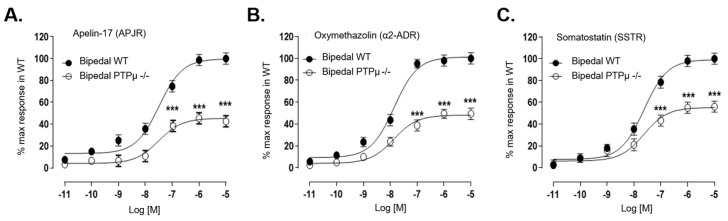
Osteoblasts from bipedal PTPµ-deficient mice demonstrate less response to GiPCR stimulation. Osteoblasts from bipedal wild-type (WT) and PTPµ −/− mice were stimulated with increasing concentrations of Apelin-17 (**A**), oxymethazolin (**B**), or somatostatin (**C**). Concentration–response curves were generated with Prism software (version 6.03) using data normalized to the response achieved at maximal stimulation in osteoblasts from bipedal WT mice. (*** *p* > 0.001, *t*-test). Data are expressed as mean ± standard error of the mean of experiments performed three times in duplicate for at least 12 mice per genotype.

We previously demonstrated a systemic Gi-coupled receptor signaling dysfunction in osteoblasts and other cell types derived from AIS patients [14]. To explore whether PTPµ deficiency contributes to this defect, we analyzed osteoblasts from both WT and *Ptprm*-null mice in response to three Gi-coupled receptor selective agonists: apelin-17, oxymetazoline, and somatostatin (Figure 3). In line with our previous findings with human osteoblasts, all three agonists produced typical signaling responses in WT osteoblasts, which were inhibited by pertussis toxin (PTX) pre-treatment, confirming Gi protein coupling for these receptors. Both WT and *Ptprm*-null osteoblasts showed concentration-dependent signaling responses to the agonists (Figure 3A–C). However, *Ptprm*-null osteoblasts exhibited reduced responsiveness compared to WT, despite similar EC50 values in both groups (Supplementary Table S7). This indicates that PTPµ deficiency affects Gi protein function regardless of the specific Gi-coupled receptor.

**Figure 4:**
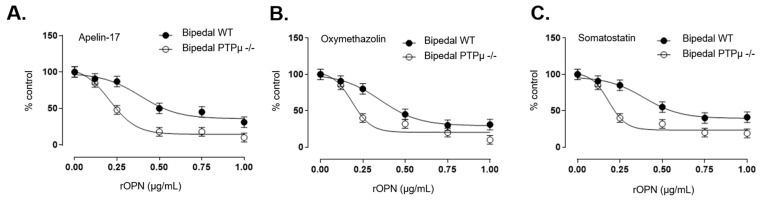
Osteoblasts from bipedal PTPµ-deficient mice demonstrate less response to GiPCR stimulation following rOPN treatment. Osteoblasts from bipedal wild-type (WT) and PTPµ −/− mice were treated with recombinant OPN (rOPN) before Gi-coupled receptor stimulation with Apelin-17 (**A**), oxymethazolin (**B**), or somatostatin (**C**). Concentration–response curves were generated with Prism software using data normalized to the response achieved at maximal stimulation in osteoblasts from bipedal WT mice. Data are expressed as mean ± standard error of the mean of experiments performed three times in duplicate for at least 12 mice per genotype.

To assess whether OPN mediates this effect, we treated WT and *Ptprm*-null osteoblasts with varying concentrations of recombinant OPN (rOPN) before stimulating them with three different Gi-coupled receptor agonists (Figure 4A–C). Pre-treatment with rOPN caused a concentration-dependent reduction in signaling responses in both WT and *Ptprm*-null osteoblasts. However, the IC50 values were lower in *Ptprm*-null cells, suggesting that these osteoblasts were more sensitive to OPN’s inhibitory effects on Gi-coupled receptor signaling (Supplementary Table S8).

**Figure 5:**
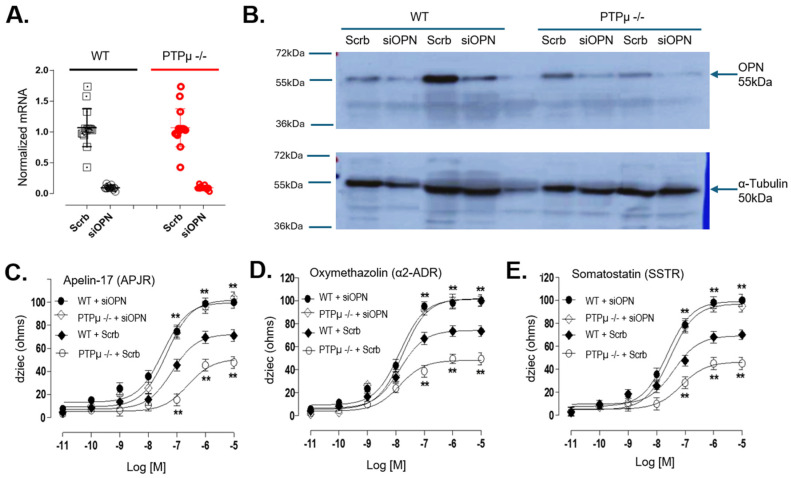
OPN silencing rescues Gi-coupled receptor signaling impairment in PTPµ −/− osteoblast. Osteoblasts from bipedal wild-type (WT) and PTPµ −/− mice were transfected with scramble (Scrb) or osteopontin (OPN) siRNA (siOPN) and efficiency of siRNA was verified with qPCR 48 h after transfection (n = 12 per genotype) (**A**) and by Western blot (n = 2 per genotype) (**B**) using α-Tubulin as the internal control. The effect of OPN silencing on Gi-coupled receptor signaling was evaluated by cellular dielectric spectroscopy by treating mouse osteoblasts with increasing concentrations of Apelin-17 (**C**), oxymethazolin (**D**), or somatostatin (**E**). Concentration–response curves were generated with Prism software. Symbols: open circle = PTPµ −/− mice transfected with Scrb; closed circle = wild-type (WT) mice transfected with siOPN; open diamond = PTPµ −/− mice transfected with siOPN and closed diamond = wild-type (WT) mice transfected with Scrb. Data are expressed as mean ± standard error of the mean of experiments performed three times in duplicate for at least 12 mice per genotype. * *p* < 0.05, ** *p* < 0.01 versus Scrb-transfected WT group based on one-way ANOVA, then followed by the Dunnett’s post doc test.

Since OPN is naturally expressed in both mouse and human osteoblasts, we used small interfering RNA (siRNA) to knock down *Spp1* gene expression, reducing endogenous OPN levels in both WT and *Ptprm*-null osteoblasts. The knockdown efficiency was confirmed at the mRNA level via qPCR (Figure 5A) and at the protein level by Western blot (Figure 5B). Silencing the *Spp1* gene to lower OPN levels enhanced Gi-coupled receptor signaling in WT mouse osteoblasts in response to selective ligands, each of which elicited signaling through distinct Gi protein-coupled receptors. This approach also rescued the impaired Gi-coupled receptor signaling in *Ptprm*-null osteoblasts, eliminating the signaling differences between the two groups (Figure 5C–E).

### 2.7. OPN Preferentially Interacts with α5β1 Integrin in PTPµ −/− Osteoblasts

**Figure 6:**
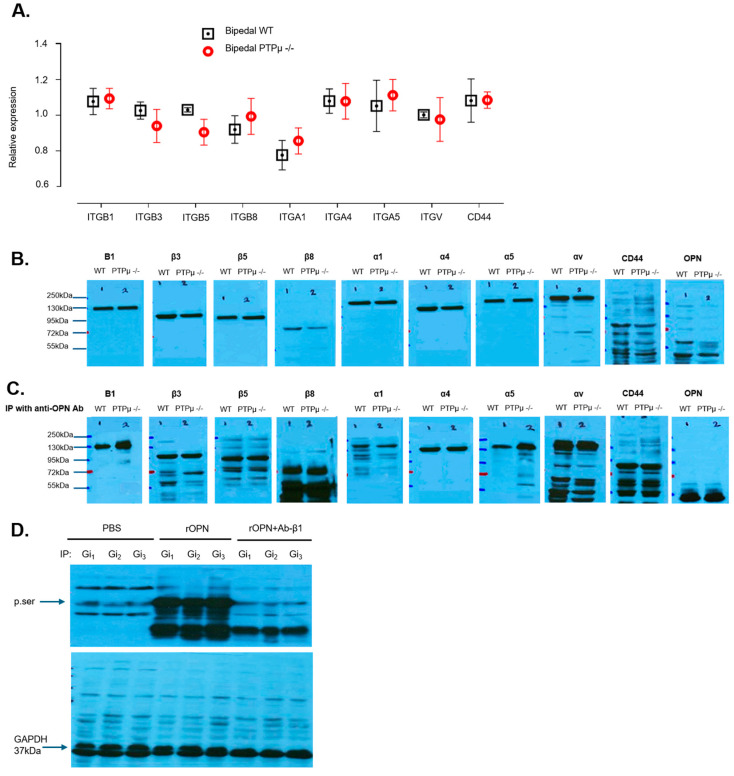
Loss of PTPµ contributes to increase the affinity of α5β1 integrin toward OPN. (**A**) Total RNA was extracted from osteoblasts of bipedal wild-type (WT) and PTPµ −/− mice, and mRNA expression levels of CD44 and indicated beta (β) alpha (α) integrins were examined by qPCR analysis using β-actin as the internal control. Error bars show standard error of the mean of three independent experiments performed in duplicate. (**B**) WT and PTPµ −/− osteoblasts (samples from 3 mice per genotype were pooled (n = 3)) were lysed and 20 µg of proteins were resolved by 10% SDS-PAGE and immunoblotted for antibodies specific for the indicated proteins. OPN served as the loading control. (**C**) WT and PTPµ −/− osteoblasts were lysed and subjected to immunoprecipitation (samples from 3 mice per genotype were pooled (n = 3)) with antibodies directed against each integrin subunit as indicated, including CD44 and OPN, and immunoprecipitates were resolved by 10% SDS-PAGE. Western blots were revealed with an antibody raised against OPN. OPN served as the loading control. The molecular weights for the integrins β1, β3, β5, β8, α1, α4, α5, αv, CD44, OPN are respectively 138kDa, 125kDa, 100kDa, 97kDa, 150kDa, 150kDa, 150kDa, 135kDa, 81kDa and 66kDa. (**D**) MC3T3-E1 cells (mouse osteoblasts) treated with PBS or rOPN were subjected to immunoprecipitation with antibodies against the Gi_1_, Gi_2_, or Gi_3_ alpha subunit. Precipitates were resolved by 10% SDS-PAGE and immunoblotted with an antibody directed against phospho-serine. Cells were pre-treated with antibody against β1 integrin for 30 min followed by an 18 h incubation with 0.5 µg/mL rOPN, prior to immunoprecipitation and immunoblotting. Bands shown are representative of results obtained with independent experiments. A second replicative experiment is shown in Supplementary Figure S6.

Since osteoblasts express a variety of receptors that can interact with OPN such as integrin αvβ1, αvβ3, αvβ5, α4β1, α5β1, α8β1, and CD44 receptor [22,23,24], we aimed to determine whether the systemic impairment of Gi-coupled receptor signaling mediated by OPN is specifically linked to one of these receptors. To investigate this, we first conducted qPCR analysis to evaluate the expression levels of these receptors. Our results showed no significant differences between WT and PTPµ −/− osteoblasts (Figure 6A), with both groups displaying comparable mRNA levels for each receptor. Similarly, protein levels, as assessed by Western blot, were consistent between the two groups (Figure 6B). These findings indicate that PTPµ deficiency does not impact the expression of known OPN receptors.

Next, we explored whether PTPµ deficiency affects the physical interaction of OPN with these receptors. We immunoprecipitated cell lysates from WT and PTPµ −/− osteoblasts using an anti-OPN antibody, and then detected for possible changes among the receptors interacting with OPN by Western blot (Figure 6C). Interestingly, the levels of β1 and α5 integrins in OPN immunoprecipitates were more than 30-fold higher in PTPµ −/− osteoblasts compared to WT osteoblasts, whereas the levels of other integrins showed only a slight increase (0.8- to 2.3-fold) between the two groups. In contrast, the levels of CD44 in OPN immunoprecipitates were similar in both WT and PTPµ −/− osteoblasts. Moreover, treating MC3T3-E1 cells (mouse osteoblasts) with recombinant OPN (rOPN) induced serine phosphorylation of Gi protein alpha subunits, consistent with our previous findings in AIS osteoblasts [14] (Figure 6D). Pre-treatment with an anti-β1 integrin antibody blocked this effect. Collectively, these results demonstrate that OPN plays a crucial role in the Gi-coupled receptor signaling dysfunction observed in AIS patients [14], and that PTPµ deficiency exacerbates OPN’s inhibitory effect on this pathway through its preferential interaction with the α5β1 integrin.

### 2.8. Silencing of PIPK1γ90 Rescues Gi Signaling Impairment in PTPµ −/− Osteoblasts

To explore the molecular basis for the preferential interaction of OPN with α5β1 integrin in the absence of PTPµ, we focused on Phosphatidylinositol-4-Phosphate 5-Kinase Type-I gamma (encoded by the *PIP5K1C* gene). This was based on the critical role of tyrosine-phosphorylated PIPK1γ90 in enhancing integrin–ligand affinity and its known regulation by PTPµ-mediated dephosphorylation [25]. PIPK1γ90, an isoform of *PIP5K1C*, is essential for processes such as cytoskeletal organization, cell adhesion, and signal transduction. Given that PTPµ normally dephosphorylates PIPK1γ90, we hypothesized that the loss of PTPµ would lead to sustained phosphorylation of PIPK1γ90, amplifying the Gi-coupled receptor signaling dysfunction induced by OPN.

**Figure 7:**
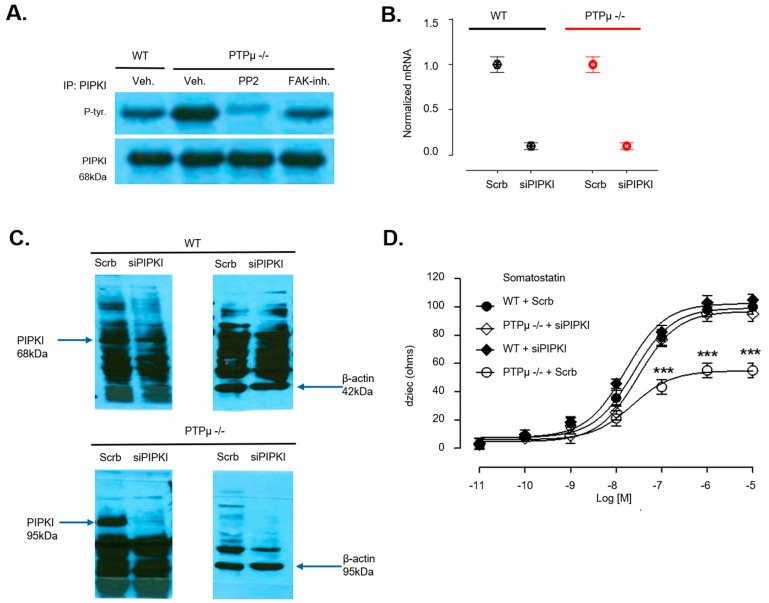
PIPKIγ90 is deregulated in osteoblasts from PTPµ −/− mice. (**A**) Cell lysates were prepared with osteoblasts from WT and PTPµ −/− mice and proteins subjected to immunoprecipitation with anti-PIPKIγ90 antibody. Immunoprecipitates were resolved by 10% SDS-PAGE and immunoblotted with an antibody directed against phospho-tyrosine or PIPKIγ90 as the control band (n = 1 per genotype). Data shown is a representation, of a replicated experiment. (**B**) Osteoblasts from WT and PTPµ −/− mice were transfected with scramble (Scrb) or OPN siRNA and efficiency of siRNA was verified with qPCR 48 h after transfection (n = 3) and by Western blot (samples from 3 mice per genotype were pooled (n = 3)) (**C**), with β-actin serving as the loading control. (**D**) Effect of PIPKIγ90 depletion on impedance signature of Gi-coupled receptor signaling was evaluated by cellular dielectric spectroscopy by challenging WT and PTPµ −/− osteoblasts with 10 µM somatostatin and analyzed with prism software. Data are expressed as mean ± SEM of experiments performed three times in duplicate for at least 12 mice per genotype. *** *p* < 0.001, versus (WT + Scrb) based on one-way ANOVA followed by Dunnett’s post doc test.

To investigate this, we examined the phosphorylation status of PIPK1γ90 in both WT and PTPµ −/− osteoblasts. Cell lysates were immunoprecipitated using a PIPK1γ90 antibody and then probed with a phospho-tyrosine antibody. As expected, PTPµ −/− osteoblasts exhibited higher levels of phosphorylated PIPK1γ90 compared to WT osteoblasts, while total PIPK1γ90 levels remained unchanged between the groups (Figure 7A). This suggests that the absence of PTPµ results in sustained activation of PIPK1γ90, thereby enhancing OPN inhibitory effect on Gi-coupled receptor signaling by increasing integrin–ligand affinity.

We hypothesized that FAK (focal adhesion kinase) and Src (proto-oncogene tyrosine-protein kinase) activation resulted in the increased phosphorylation of PIPK1γ90 in PTPµ −/− osteoblasts. Both kinases were selected based on previous studies that identified them as direct regulators of PIPK1γ90 phosphorylation [26,27]. FAK and Src have been shown to phosphorylate PIPK1γ90 on tyrosine residue, which enhances its activity in integrin-mediated signaling pathways. Additionally, PTPµ has been reported to counteract the activity of FAK and Src by dephosphorylating their substrates, thereby modulating integrin–ligand interactions. Given this evidence, we hypothesized that the loss of PTPµ would lead to unchecked FAK and Src activity, resulting in elevated PIPK1γ90 phosphorylation.

Next, we explored the role of PIPK1γ90 in the mechanism of PTPµ deficiency by knocking down PIPK1γ90 expression using siRNA in both WT and PTPµ −/− osteoblasts. The siRNA efficiency was confirmed by qPCR (Figure 7B) and Western blot analysis (Figure 7C). Varying concentrations of somatostatin were applied to WT or PTPµ −/− osteoblasts; PIPK1γ90 depletion enhanced the response in PTPµ −/− osteoblasts but not in WT cells. Additionally, PTPµ −/− osteoblasts depleted of PIPK1γ90 responded similarly to somatostatin as WT osteoblasts, indicating that the absence of PIPK1γ90 eliminates the differences in Gi-coupled receptor signaling between the two genotypes (Figure 7D). Overall, these findings suggest that the amplified dysfunction in Gi-coupled receptor signaling observed in PTPµ −/− osteoblasts is driven by increased PIPK1γ90 activity, resulting from the loss of PTPµ.

## 3. Discussion

The etiology of AIS remains complex and multifactorial, involving genetic and epigenetic factors. In this study, we focused on protein tyrosine phosphatase µ (PTPµ), encoded by the *PTPRM* gene, to explore its role in AIS pathogenesis and spinal deformity progression. Our findings demonstrated a significant downregulation of *PTPRM* gene expression and corresponding PTPµ protein levels in AIS osteoblasts from severe cases, compared to non-scoliotic trauma controls. While three rare variants were identified in the *PTPRM* gene, these variants did not significantly correlate with AIS severity or susceptibility, prompting us to investigate epigenetic mechanisms. Through this approach, we discovered five circulating miRNAs (miR-103a-3p, miR-107, miR-148a-3p, miR-148b-3p, and miR-152-3p) predicted to target *PTPRM*. These miRNAs were significantly upregulated in AIS patients with severe spinal deformities, with miR-148b-3p showing a particularly marked elevation in osteoblasts from these patients. ROC curve analysis demonstrated the prognostic potential of this miRNA panel, which could accurately differentiate symptomatic patients at risk of developing severe spinal deformities (Cobb angle ≥ 45°) from those likely to develop only mild-to-moderate scoliosis (Cobb angle 10°–44°) at skeletal maturity. Notably, miR-148b-3p is known or predicted to target genes such as *ATP2B4* and *CHD7*, both implicated in AIS pathogenesis [28,29]. For instance, *ATP2B4* encodes the plasma membrane calcium ATPase (PMCA4b), a protein previously reported to be decreased in osteoblasts from severe AIS patients within our French-Canadian cohort [28]. Interestingly, *ATP2B4* (PMCA4B) is also a predicted target of miR-96-5p, previously found to be upregulated in plasma and osteoblast samples from AIS patients in Chinese and French-Canadian populations [30]. The elevation of miR-96-5p expression was further linked to low bone mass in Chinese AIS patients [30]. Despite the identification of miR-148b-3p and other miRNAs targeting *PTPRM*, none of these miRNAs were reported in previous studies exploring circulating miRNAs as biomarkers for AIS and spinal deformity progression [31,32,33,34,35,36,37,38,39,40].

The lack of reproducibility between studies can be attributed to several factors. Firstly, the genetic and environmental heterogeneity among different populations, can lead to variability in miRNA expression. Secondly, differences in study design, sample size, biological sexes, as well as methodologies can affect the reproducibility of results. Worth mentioning is that we measured our circulating miRNAs in much younger AIS patients (mean age of 13.2 years) exhibiting less severe scoliosis (mean Cobb angle of 26°) and a Risser index < 2, while the other studies investigated much older patients (10–18 years old) with more advanced scoliosis (often a Cobb angle ≥ 40° and a Risser index ≥ 2). Lastly, the complex regulatory networks involving miRNAs mean that their effects can be context-dependent, influenced by various biological factors (e.g., sexual hormones). These challenges highlight the need for standardized protocols and larger, more diverse cohorts to validate findings and ensure their applicability across different populations.

The functional role of PTPµ deficiency in spinal deformity progression was further elucidated using a combination of in vivo and in vitro models, particularly through the use of *Ptprm*-null mice. While these mice did not spontaneously develop scoliosis, the incidence and severity of scoliosis increased significantly following amputation of the forelimbs and tail to induce scoliosis. This suggests that PTPµ acts as a modifier of spinal deformity progression, rather than an initiator of the disease. Additionally, our in vitro experiments revealed that scoliosis exacerbation in *Ptprm*-null mice is associated with increased dysfunction in OPN-mediated Gi-coupled receptor signaling.

Building on prior work by Akoume et al., which established OPN’s role in systemic Gi-coupled receptor signaling impairments in AIS patients [14], our findings demonstrated that OPN inhibits Gi-coupled receptor signaling via its preferential interaction with α5β1 integrin. This pathway is finely regulated by talin binding, which activates integrins, enabling their interaction with ligands such as OPN. A key step in this process is the phosphorylation of PIPK1γ90, an enzyme that generates PI4,5P2 and promotes talin–integrin binding. Our data show that PTPµ plays a critical role in maintaining PIPK1γ90 in a hypophosphorylated state, limiting excessive α5β1 integrin activation by OPN. Conversely, PTPµ depletion results in hyperphosphorylation of PIPK1γ90, allowing OPN to preferentially bind α5β1 integrin. This disrupts normal cellular signaling and potentially competes with other extracellular matrix components such as fibronectin and laminin. This shift in integrin binding affinity likely contributes to the increased severity of spinal deformities observed in AIS patients with reduced PTPµ expression.

Despite promising findings, some limitations exist in this study. The small sample size of the genetic association study may have limited the ability to detect significant associations between rare variants in *PTPRM* and scoliosis severity. Larger, multiethnic cohorts are needed to validate these findings and uncover additional genetic variants contributing to AIS. Furthermore, while miRNA expression profiles and functional assays highlighted miR-148b-3p’s role in regulating *PTPRM*, further in vivo studies are required to confirm its inhibitory effect in the reduction in PTPµ protein in AIS osteoblasts and other relevant tissues like the paraspinal muscles.

In summary, this study underscores the importance of PTPµ in modulating scoliosis severity in AIS. PTPµ reduction, potentially driven by epigenetic regulation via miRNAs like miR-148b-3p, exacerbates spinal deformity progression by enhancing OPN-α5β1 integrin interactions. In this context, the identification of OPN-α5β1 integrin interaction as a key driver of scoliosis progression highlights the potential for therapeutic interventions targeting this pathway. Further studies are needed to explore the clinical applicability of these approaches to mitigate, reverse, or prevent scoliosis in the near future.

## 4. Materials and Methods

### 4.1. Sex as a Biological Variable

Though AIS has a significant prevalence among females, both female and male subjects were studied within both the human and mouse studies, though this led to the skewed sample size (more female patients). Controls were matched for age and sex in all cases.

### 4.2. Study Populations

French-Canadian patients with adolescent idiopathic scoliosis (AIS, N = 804) and healthy controls matched by age and sex (N = 239) were recruited from three pediatric spine centers in Montreal and local schools (Table 1 and Supplementary Tables S1 and S4). All participants were of Caucasian and European descent and underwent a clinical examination by an orthopedic surgeon. Healthy controls were screened using Adam’s forward-bending test and a scoliometer, excluding children with any visible spinal curvature or a family history of scoliosis. Detailed medical histories were obtained from all participants, and no additional health conditions were reported during sample collection. Ancestry and genetic relationships were assessed using the EIGENSTRAT and PLINK’s identity-by-descent methods (accessed on 25 July 2013). AIS patients were classified based on the severity of their spinal deformities, with mild-to-moderate cases defined by a Cobb angle between 10° and 44°, and severe cases by a Cobb angle of 45° or greater.

### 4.3. Patients and Specimens

Plasma samples were collected from a subset of participants: 36 severe AIS cases (progressors), 28 non-severe AIS cases (non-progressors), and 28 healthy controls. Detailed clinical and demographic information for each subgroup is provided in Supplementary Table S1. Osteoblast samples were obtained from 11 severe AIS surgical cases and 3 non-scoliotic trauma cases as controls (Supplementary Table S2). This comprehensive study design facilitated the investigation of PTPµ and microRNA involvement in AIS pathogenesis and their potential as biomarkers for disease progression. Approximately 10 mL of peripheral blood was obtained from each of the control subjects and AIS patients, and bone specimens were from vertebras of AIS and from the tibia or femur of non-scoliotic patients. In AIS patients, bone specimens were obtained intra-operatively from vertebrae varying from T3 to L4. Written consent was obtained for each participant from the parents or legal guardians while participants gave their assents. This study and protocol were approved by the Institutional Review Board of each participating institution. All experiments were performed following relevant guidelines and human ethics regulations. The study was approved by the Institutional Review Board of CHU Sainte-Justine (Comité D’Éthique CHU Sainte-Justine, Project # 2007–120, initial approval date May 2002); the McGill Institutional Review Board, for the studies conducted at The Montreal Children’s Hospital and The Shriners Hospital for Children (Project # A02-M15-03B, initial approval date September 2003)*;* the English Montreal School Board Research Committee (Project # 2380, initial approval date September 2007); and the Research Committee of The Affluent School Board (Comité de recherche de la Commission Scolaire Des Affluents, Project # CSA2380, initial approval date September 2003).

### 4.4. Animals

Breeding pairs of PTPµ knockout mice on the CB57BL/6 background were a generous gift from Dr. Gebbink MF (University Medical Center Utrecht, Utrecht, The Netherlands), and the CB57BL/6 wild-type mice were purchased from Charles River Laboratories (St. Constant, QC, Canada). The animals were bred as separate colonies in a pathogen-free facility maintained at 25 °C on a 12 h light/dark cycle. Each animal had free access to food and water. Bipedal conditions were induced in 4-week-old mice by amputation of the forelimbs and tails under anesthesia, as previously described [11,12]. Bipedal mice were screened for scoliosis at 12-, 24-, and 36-weeks post-surgery, using a Faxitron X-rays apparatus (Faxitron X-rays Corp., Wheeling, IL, USA). All procedures involving animals were conducted in accordance with the guidelines of the Canadian Council on Animal Care (CCAC) and approved by the Institutional Animal Care and Use Committee of Sainte-Justine University Hospital (CHU Sainte-Justine, protocol #336, initial approval date October 2008).

### 4.5. Mouse Blood Collection and OPN Measurement

Peripheral blood from anesthetized mice was collected at 12-, 24-, and 36-weeks post-surgery into EDTA-treated tubes, and plasma was then separated by centrifugation at 1000× *g* for 10 min at 4 °C and stored at −80 °C. Plasma OPN protein concentrations were measured using a specific ELISA kit from IBL International (Hamburg, Germany) according to the manufacturer’s instructions. All OPN measurements were performed in duplicate and absorbance was measured using a DTX 880 Multimode Detector (Beckman Coulter, Brea, CA, USA).

### 4.6. Cell Culture

Osteoblasts were prepared as previously described [5], and maintained in α-modified minimum essential medium (α-MEM, Wisent, Saint-Bruno, QC, Canada) supplemented with 10% fetal bovine serum (FBS, Thermo Fisher Scientific, Waltham, MA, USA) and 1% penicillin/ streptomycin (Thermo Fisher Scientific) under standard conditions (37 °C/5% CO_2_). Culture media were renewed every three days, and cells were allowed to grow to 90% confluency.

### 4.7. siRNA Transfection

Cells were transiently transfected with appropriate siRNA in a serum-free medium using Lipofectamine RNAiMAX reagent (Thermo Fisher Scientific) according to the manufacturer’s instructions. The cells were harvested for RNA extraction after 48 h, and the gene knockdown was verified by quantitative real-time PCR (qPCR). siRNA for the knockdown of OPN or phosphatidylinositol-phosphate kinase type I gamma (PIPK1γ90) and scrambled siRNA were obtained from Thermo Fisher Scientific.

### 4.8. Quantitative Reverse Transcription-Polymerase Chain Reaction (qPCR)

Reverse transcription of 1 µg of mRNA was performed using Reverse Transcriptase (Thermo Fisher Scientific) and the designed human primers listed below:

OPN Forward: 5′-GTCCCCACAGTAGACACATATG-3′

OPN Reverse: 5’-TCAACTCCTCGCTTTCCATG-3’

β1 integrin Forward: 5’-TTGTCCCCGACTTTCTACCTT-3’

β1 integrin Reverse: 5’-ATGTGTCAGACCTGCCTTG-3’

β3 integrin Forward: 5’-GGAAAGTCCATCCTGTATGT-3’

β3 integrin Reverse: 5’-GAGTTTCCAGATGAGCAGGG-3’

β5 integrin Forward: 5’-CTTGCACTCCTGGCTATCTG-3’

β5 integrin Reverse: 5’-TGCGTGGAGATAGGCTTTC-3’

β8 integrin Forward: 5’-GATTGGGTTGCTTAAAGTCC-3’

β8 integrin Reverse: 5’-GGTAGGTGACTGCTTCTTGTG-3’

α1 integrin Forward: 5’-GACATTTGGATCAACTTTAG-3’

α1 integrin Reverse: 5’-GGCAATGGAATTCACGACTT-3’

α4 integrin Forward: 5’-GGATGAGACTTCAGCACTCA-3’

α4 integrin Reverse: 5’-GGTGAAATAACGTTTGGGTC-3’

α5 integrin Forward: 5′-CCAGGCCAGTTCCATCTATG-3′

α5 integrin Reverse: 5′-ATGTCTGAGCCATTAAGGATGG-3′

αv integrin Forward: 5′ -GCTCAGTGCTTGAAGATTGT-3′

αv integrin Reverse: 5′ -GCAGACGAACTTCAGAGAATA-3′

CD44 Forward: 5′-AGCATCGGATTTGAGACCTG-3′

CD44 Reverse: 5′-TGAGTCCACTTGGCTTTCTG-3′

PTPµ forward 5′- GGCCGGACTTTTGCTAACT -3′

PTPµ reverse 5′- TGTGCTATACGGCTCATCAAA -3′

β-actin forward 5′-GGAAATCGTGCGTGACAT-3′,

β-actin reverse 5′-TCATGATGGAGTTGAAGGTAGTT-3′

The qPCR reaction was performed in a total volume of 25 μL using QuantiTect SYBR Green PCR Master Mix (QIAGEN Inc., Hilden, Germany) and performed on the Stratagene Mx3000P system (Agilent Technologies, La Jolla, CA, USA). Each reaction consisted of 5 μL of cDNA (diluted 1:10 or 1:40, depending on the target gene), 12.5 μL of 2× QuantiTect SYBR Green PCR Master Mix, and 7.5 μL of primer solution (containing both forward and reverse primers). The qPCR was performed in duplicate for each sample. The amplification data were analyzed with the MxPro QPCR Software, version 2.0. Relative quantification was calculated using the delta cycle thresholds (ΔCT) method, with β-actin as the endogenous control.

### 4.9. Immunoprecipitation and Western Blot

Cells were lysed in RIPA buffer (25 mM Tris.HCl pH 7.4, 150 mM NaCl, 1% NP-40, 1% sodium deoxycholate, 0.1% SDS) containing 5 mM NaVO_4_ and protease inhibitor cocktail (Roche Molecular Biochemicals, Mannheim, Germany). For immunoprecipitation, lysates were first pre-cleared with 25 μL of protein Sepharose A beads (GE Healthcare Bioscience, Piscataway, NJ, USA). The supernatant was then incubated with appropriate antibodies, followed by 1 h incubation with protein G beads with gentle rocking. The beads were washed three times with lysis buffer, and bound proteins were eluted with gel loading dye and boiled at 100 °C for 5 min before being separated on 10% SDS-PAGE and blotted to nitrocellulose. The supernatant was then incubated with anti-PTPµ antibody (SC-25433), (Santa Cruz Biotechnology Inc., Dallas, TX, USA) or anti- OPN antibody (sc10593; Santa Cruz Biotechnology, Inc.), followed by 1 h incubation with protein A beads with gentle rocking. The beads were washed three times with lysis buffer, and bound proteins were eluted with gel loading dye and boiled at 100 °C for 5 min before being separated on 10% SDS-PAGE and blotted to nitrocellulose. The immune complexes were analyzed by Western blot analysis with antibody against integrin β1 (SC-6622), integrin β3 (SC-6627), integrin β5 (SC-5401), integrin β8 (SC-514150), integrin α1 (sc-271034), integrin α4 (sc-6589), integrin α5 (sc-166681), (Santa Cruz Biotechnology Inc.), or integrin αv (4711) (Cell Signaling Technology, Danvers, MA, USA). Immunoreactivity was visualized using the SuperSignal West Pico Chemiluminescent Substrate kit (Pierce Biotechnology Inc., Rockford, IL, USA).

### 4.10. Cellular Dielectric Spectroscopy (CDS) Assay

A CDS assay was performed using the CellKey^TM^ apparatus (Molecular Devices, San Jose, CA, USA) as previously described [7,8]. In brief, cells were seeded into the CellKey^TM^ standard 96-well microplate at a density of 10 × 10^4^ cells per well and incubated in standard conditions (37 °C/5% CO_2_). After overnight incubation, cells were either directly stimulated with apelin-17, oxymethazolin, or somatostatin (Tocris Chemical Co. St. Louis, MO, USA) or treated with 0.5 µg/mL exogenous recombinant OPN (rOPN) for 18 h prior stimulation. For experiments with Pertussis toxin (PTX), cells were pre-incubated with PTX (100 ng/mL, Sigma, Oakville, ON, Canada) for 16 h before stimulation.

### 4.11. miRNA Extraction and qPCR

miRNA extraction from human plasma and osteoblasts was performed using the mirVana PARIS RNA and Native Protein Purification Kit (Thermo Fisher Scientific) according to the manufacturer’s instructions. For plasma samples, 50 nmol of the synthetic oligonucleotide Cel-miR-39-3p RNA (UCACCGGGUGUAAAUCAGCUUG, Thermo Fisher Scientific) was added as the exogenous control, while miR-16-5p served as the internal control for osteoblasts, given its stable expression across different cell types, including osteoblasts. cDNA synthesis was carried out using the TaqMan Advanced miRNA cDNA Synthesis Kit (Thermo Fisher Scientific) following the manufacturer’s protocol. The qPCR reaction was conducted in duplicate using TaqMan Advanced miRNA Assays for each target miRNA (Thermo Fisher Scientific) and analyzed on the QuantStudio 3 instrument (Thermo Fisher Scientific). Normalization was performed using the CT values of the exogenous control (Cel-miR-39a-3p) for plasma or the internal control (miR-16-5p) for osteoblasts: ΔCTsample = CTmiR − CTexogenous/endogenous control. The fold change in miRNA expression was calculated using the 2^−ΔΔCT^ method. To identify potential target genes and molecular functions, Ingenuity Pathway Analysis (IPA) software (QIAGEN Inc., Hilden, Germany, software version 51963813) was used.

### 4.12. Statistics

Data are presented as mean ± standard deviation (SD) and were analyzed by ANOVA or Student’s *t*-test using GraphPad Prism 4.0 software. Multiple comparisons of means were performed with one-way ANOVA followed by Dunnett’s post hoc test. For the statistical analysis of miRNAs, multiple comparisons of means were performed with one-way ANOVA. Only *p*-values < 0.05 were considered significant.

## 5. Patents

This work led to a patent application (pending) owned by CHU Sainte-Justine.

## Figures and Tables

**Table 1:** Clinical and demographic characteristics of the French-Canadian cohort.

	AIS Patients (Progressor)			AIS Patients (Non-Progressors)			Healthy Control Subjects		*p*-Value (vs. Controls)
	**N**	**Age** **(Years)**	**Highest Cobb Angle °**	**N**	**Age** **(years)**	**Highest Cobb ** **Angle °**	**N**	**Age** **(Years)**	
All	111	15.8 ± 2.1 [10.9–21.5]	52.9 ± 9.1 [40–74]	147	16.8 ± 0.9 [15.2–20.1]	19.7 ± 6.3 [10–33]	143	12.5 ± 3.2 [3.2–18.3]	<0.001
Female	94	15.6 ± 1.9 [10.9–21.2]	54.1 ± 9.4 [43–74]	120	16.8 ± 0.9 [15.2–20.1]	20.1 ± 6.1 [10–33]	67	12.5 ± 3.3 [4.6–18.3]	<0.001
Male	17	16.8 ± 2.5 [11.9–21.5]	47.2 ± 5.4 [40–54]	27	16.8 ± 0.8 [15.6–19.2]	18 ± 7.2 [10–29]	76	12.5 ± 3.2 [3.2–17.6]	<0.001

The data are represented as mean ± standard deviation [min–max range]. An independent samples *t*-test was used to compare the ages of the progressor and non-progressor AIS groups to the healthy controls. Fisher’s exact test was used to compare the sex distributions between the AIS groups and the healthy controls. AIS = adolescent idiopathic scoliosis; N = sample size.

**Table 2:** Comparison of plasma OPN level in bipedal WT vs. PTPµ −/− mice.

Condition	Characteristics					
	Sex	Mice Genotype	N	Mean OPN Levels (ng/mL)	Range	*p*-Value
Non-Scoliotic	Male	Wild Type	4	3460 ± 193	2723–4105	0.772
	Male	PTPµ −/−	2	3713 ± 220	2586–4839	
Scoliotic	Male	Wild Type	10	4612 ± 48	3726–6094	0.284
	Male	PTPµ −/−	17	4922 ± 220	3574–5958	
Non-Scoliotic	Female	Wild Type	5	3480 ± 160	2232–4195	0.382
	Female	PTPµ −/−	5	3859 ± 185	3350–4632	
Scoliotic	Female	Wild Type	14	4420 ± 102	3352–6298	0.585
	Female	PTPµ −/−	34	4548 ± 214	3440–5954	

Data are presented as mean ± standard deviation (SD). *p*-values were calculated using the *t*-test to compare OPN levels between *Ptprm*-null and wild-type mice within each sex and condition. Values of mean plasma OPN levels were determined at 36 weeks’ post-surgery.

## Data Availability

The datasets that were generated and used for this study are available through the corresponding author upon reasonable request.

## Supplementary Materials

The following supporting information can be downloaded at: https://www.mdpi.com/article/10.3390/ijms26031042/s1.
