# Supplementary material for: Loss of Tyrosine Phosphatase Mu Promotes Scoliosis Progression Through Osteopontin-α5β1 Integrin Signaling and PIPK1γ90 Activity
Source: Int J Mol Sci. 2025 Jan 26;26(3):1042. doi: 10.3390/ijms26031042 (PMC11816665; doi:10.3390/ijms26031042)
Supplement: Supplementary file 1 [file ijms-26-01042-s001.zip › ijms-3381856-supplementary.pdf]

Loss of Tyrosine Phosphatase Mu Promotes Scoliosis Progression Through Osteopontin-$\alpha5\beta1$ Integrin Signaling and PIPK1γ90 Activity

Mohamed Elbakry [1,2,†], Nasrin Khatami [1,3,†], Marie-Yvonne Akoume [1,4,*], Cédric Julien [1], Saadallah Bouhanik [1], Anita Franco [1], Iurie Caraus [1], Wesam Elremaly [1] and Alain Moreau [1,4,5,*]

1 Viscogliosi Laboratory in Molecular Genetics of Musculoskeletal Diseases, Azrieli Research Center, CHU Sainte-Justine, Montréal, QC, H3T 1C5, Canada; mohamed.elbakry.hsj@ssss.gouv.qc.ca (M.E.); nasrin.khatami@umontreal.ca (N.K.); drick.julien@gmail.com (C.J.); bouhanik@yahoo.com (S.B.); anita.franco.hsj@ssss.gouv.qc.ca (A.F.); iurie.caraus.hsj@ssss.gouv.qc.ca (I.C.); wesam.elremaly.hsj@ssss.gouv.qc.ca (W.E.)
2 Department of Chemistry, Biochemistry Section, Faculty of Science, Tanta University, Tanta, Gharbia Governorate, Egypt
3 Department of Biochemistry and Molecular Medicine, Faculty of Medicine, Université de Montréal, Montréal, QC, H3T 1J4, Canada
4 Department of Cellular, Molecular Biology and Genetics, Faculty of Medicine, Université des Sciences de la Santé (USS) de Libreville, Libreville, Gabon
5 Department of Stomatology, Faculty of Dentistry, Université de Montréal, Montréal, QC, H3T 1J4, Canada
* Correspondence: my.akoume@gmail.com (M.-Y.A.); alain.moreau.hsj@ssss.gouv.qc.ca (A.M.); Tel.: +514-345-4931 (ext. 2667) (M.-Y.A.); +514-345-4931 (ext. 5722) (A.M.)
† These authors should be considered as co-first authors.

Supplementary Table S1. Clinical and demographic characteristics of participants used for validation of circulating microRNAs targeting *PTPRM*

Group	N	Age (years)	Sex (F/M)	*p*-value (vs. Controls)
AIS (Progressor)	36	12.9 ± 1.3	27F / 9M	Age: 0.64<br>Sex: 0.11
AIS (non-progressor)	28	13.5 ± 1.6	16F / 12M	Age: 0.11<br>Sex: 1.0
Healthy control	28	12.7 ± 2.1	15F / 13M	-

Data are presented as mean ± standard deviation (SD). *p*-values were calculated using an independent samples t-test for age comparisons and Fisher's exact test for sex comparisons between each AIS group (progressors and non-progressors) and the healthy controls.

Supplementary Table S2. Clinical and demographic characteristics of surgical AIS cases and non-scoliotic trauma control cases used for validation of osteoblast microRNAs targeting *PTPRM*

	AIS (surgical cases)	Non-scoliotic controls (trauma cases)	*p*-value
**N**	11	3	-
**Age (year)**	14.5 ± 1.4	15.1 ± 3.6	0.644
**Sex**	F	F	-
**Mean Cobb angle °**	59.7 ± 10.5	-	-

Data are presented as mean ± standard deviation (SD). An independent samples t-test was used to compare the ages of the AIS and control groups. AIS, adolescent idiopathic scoliosis.

Supplementary Table S3. List of rare variants in *PTPRM* gene detected in AIS patients

chr	Position (bp)	Ref Allele	Alt allele	Gene	Ctrls count	% ctrl	Severe AIS count	% Severe AIS	SNP ID	AA substitution	ESP6500 EA MAF	1000G Eur MAF
chr18	7774190	T	A	PTPRM	1	0,016	1	0,02	rs35224276	S39R	0,000698	0,004
chr18	8380394	T	C	PTPRM	0	0	1	0,02	unknown	V1070A	unknown	unknown
chr18	8384601	G	A	PTPRM	0	0	1	0,02	rs368817148	G1095S	0,000116	unknown

Only rare variants with a MAF lower than 2% according to public databases like ESP6500 or 1000 Genome were selected.

Supplementary Table S4. Association of *PTPRM* genotypes with AIS and scoliosis severity

SNP number	AIS progressor (surgical cases)	AIS non-progressor	Health controls
**rs1247471646**			
N	114	147	132
CC	114 (100%)	147 (100%)	132 (100%)
**rs763119679**			
N	117	149	133
TT	117 (100%)	149 (100%)	133 (100%)
**rs766403029**			
N	114	147	130
CC	114 (100%)	147 (100%)	130 (100%)
**rs368817148**			
N	114	144	137
CC	114 (100%)	144 (100%)	137 (100%)
**rs1212955204**			
N	113	134	128
CC	113 (100%)	134 (100%)	128 (100%)
**rs35224276**			
N	114	145	136
TT	112 (98.2%)	143 (98.6%)	134 (98.5%)
AT	2 (1.8%)	2 (1.4%)	2 (1.5%)

Variants rs35224276 and rs1247471646 are located in the 5' UTR and intronic regions, respectively, where they might impact *PTPRM* gene transcription or mRNA stability. Two SNPs, rs763119679 and rs766403029 are situated near splice sites, and their presence could lead to alternative splicing events or unstable mRNA transcripts, potentially reducing PTPμ protein levels. Finally, two other SNPs, rs368817148 and rs12112955204 are missense variants, which could alter the PTPμ protein structure, affecting its stability, activity, or interactions with other molecules. Changes in the protein structure could also lead to enhanced degradation or reduced functionality.

Supplementary Table S5. List of miRNAs targeting human *PTPRM* mRNA

hsa-miR-103-3p	hsa-miR-507	hsa-miR-148b-3p	hsa-miR-449a
hsa-miR-10400-3p	hsa-miR-548av-5p	hsa-miR-152	hsa-miR-449b
hsa-miR-1179	hsa-miR-556-3p	hsa-miR-152-3p	hsa-miR-449b-5p
hsa-miR-148a-3p	hsa-miR-5681a	hsa-miR-182	hsa-miR-3660
hsa-miR-182-5p	hsa-miR-634	hsa-miR-196a	hsa-miR-4526
hsa-miR-196a-3p	hsa-miR-635	hsa-miR-196a-3p	hsa-miR-3680
hsa-miR-19b-1-5p	hsa-miR-6721-5p	hsa-miR-19a	hsa-miR-3680-3p
hsa-miR-205-5p	hsa-miR-6809-5p	hsa-miR-19a-5p	hsa-miR-452-3p
hsa-miR-218-5p	hsa-miR-6838-3p	hsa-miR-19b-1	hsa-miR-557
hsa-miR-221-3p	hsa-miR-6861-5p	hsa-miR-19b-1-5p	hsa-miR-8054
hsa-miR-34a-5p	hsa-miR-7153-3p	hsa-miR-19b-2	hsa-miR-548k
hsa-miR-3660	hsa-miR-7974	hsa-miR-19b-2-5p	hsa-miR-6774-5p
hsa-miR-3680-3p	hsa-miR-103	hsa-miR-205	hsa-miR-452
hsa-miR-4437	hsa-miR-103a	hsa-miR-205-5p	hsa-miR-466
hsa-miR-450b-5p	hsa-miR-103a-3p	hsa-miR-218	hsa-miR-6867-5p
hsa-miR-452-3p	hsa-miR-107	hsa-miR-218-5p	hsa-miR-4681
hsa-miR-4540	hsa-miR-103-1	hsa-miR-221	hsa-miR-4802-3p
hsa-miR-466	hsa-miR-103-2	hsa-miR-222	hsa-miR-488
hsa-miR-466d-5p	hsa-miR-4674	hsa-miR-222-3p	hsa-miR-491-3p
hsa-miR-4681	miR-1179	hsa-mir-172	hsa-miR-507
hsa-miR-4717-3p	hsa-miR-148	hsa-miR-34a	hsa-miR-556-3p
hsa-miR-4802-3p	hsa-miR-148a	hsa-miR-34c	hsa-miR-5681a
hsa-miR-488-3p	hsa-miR-148a/b	hsa-miR-34c-5p	hsa-miR-634
hsa-miR-491-3p	hsa-miR-148b	hsa-miR-449	hsa-miR-6809-5p
hsa-miR-7974			

Supplementary Table S6. List of circulating miRNAs upregulated in AIS patients

Name	Log_2 FC	*p*-value
hsa-miR-103a-3p	2.88	0.035
hsa-miR-107	2.07	0.045
hsa-miR-148a-3p	1.36	0.042
hsa-miR-148b-3p	2.48	0.035
hsa-miR-152-3p	1.07	0.049
hsa-miR-19b-2-5p	1.39	0.027
hsa-miR-34a-5p	1.06	0.027
hsa-miR-3680-3p	1.09	0.009
hsa-miR-548av-5p	1.09	0.026
hsa-miR-557	1.04	0.004
hsa-miR-6861-5p	1.50	0.004

This table lists circulating miRNAs that were found to be upregulated in AIS patients compared to controls. Log2 FC represents the log2 fold change in miRNA expression. *p*-values were calculated using t-test to compare miRNA expression levels between the two groups. AIS, adolescent idiopathic scoliosis.

Supplementary Table S7. Agonist EC50 comparison in PTPμ -/- vs. WT mouse osteoblasts

	**WT**	**PTPμ -/-**	***p*-value**
	EC_{50} (nM)	EC_{50} (nM)	
**Apelin-17**	32.4 ± 1.3	31.4 ± 1.6	0.63
**Oxymethazolin**	13.9 ± 0.7	13.3 ± 0.4	0.46
**Somatostatin**	24.0 ± 1.4	23.6 ± 1.2	0.83

EC_{50} were calculated from concentration response curves by non-linear regression analysis using GraphPad prism. Values are expressed as mean ± SEM of experiments performed three times in duplicate for at least 12 mice per genotype.

Supplementary Table S8. Comparison of IC50 values obtained with rOPN calculated for Apelin-17, oxymethazolin and somatostatin obtained with WT and PTPμ -/- mouse osteoblasts

	**WT**	**PTPμ -/-**	***p*-value**
	IC_{50} (nM)	IC_{50} (nM)	
**Apelin-17**	24.4 ± 1.3	16.1 ± 1.6	0.001
**Oxymethazolin**	22.5 ± 1.7	15.4 ± 1.3	0.003
**Somatostatin**	24.6 ± 2.1	15.2 ± 1.2	0.001

IC_{50} were calculated from concentration response curves by non-linear regression analysis using graphPad prism. Values are expressed as mean ± SEM of experiments performed three times in duplicate for at least 12 mice per genotype. Statistical significance was determined using the T-test (** p-value<0.01).

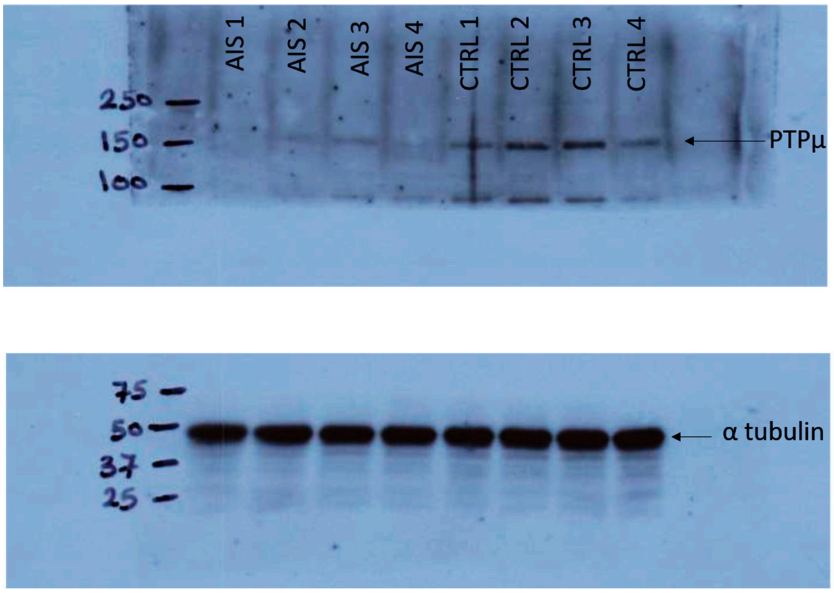

Supplementary Figure S1. Analysis of *PTPRM* protein expression in osteoblasts derived from scoliotic and non-scoliotic subjects. The Western blot shown represents a replicative experiment, demonstrating a reduction in PTPμ protein levels in osteoblasts from idiopathic scoliosis patients compared to non-scoliotic controls. PTPμ protein levels were quantified by densitometry using ImageJ software and normalized to α-Tubulin.

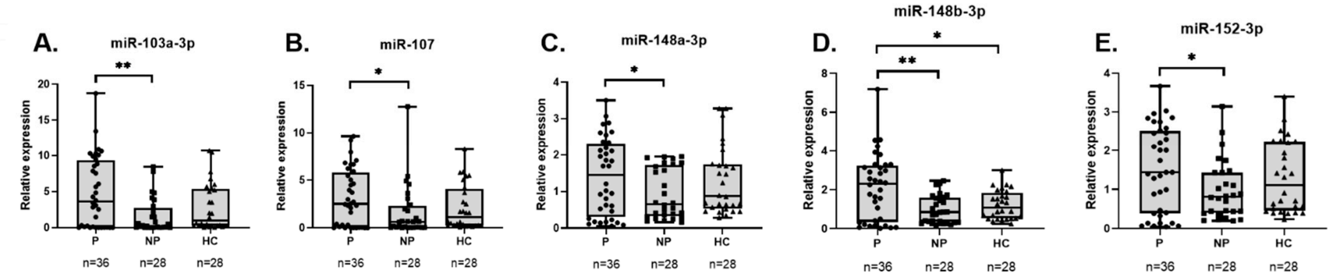

Supplementary Figure S2. Five *PTPRM*-targeting miRNAs are significantly upregulated in the plasma samples of AIS progressor compared to non-progressor cases. Panels (a-e) show the expression levels of miR-103a-3p, miR-107, miR-148a-3p, miR-148b-3p, and miR-152-3p, respectively. Relative expression levels of these circulating miRNAs were analyzed and compared across three groups: the progressor (P) group (severe AIS cases), the non-progressor (NP) group (non-severe AIS cases), and healthy controls (HC). Graphs and statistical analyses were generated in GraphPad Prism 9. Statistical significance was evaluated using one-way ANOVA for overall comparisons among the three groups, followed by post hoc tests to compare each AIS group (P and NP) with the HC group. For direct comparisons between two groups, an unpaired t-test was used. Significance levels are indicated as p-value < 0.05 and p-value < 0.01.

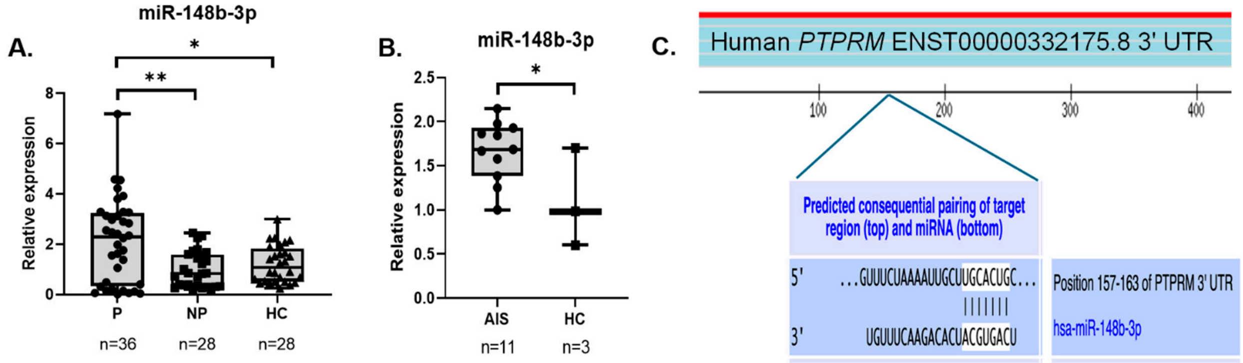

Supplementary Figure S3. Circulating miR-148b-3p is also upregulated in osteoblasts of AIS surgical cases compared to non-scoliotic controls (trauma cases). Panel a is representing the expression of miR-148b-3p in plasma samples of AIS severe or progressor group (P) and AIS non-severe or non-progressor group (NP) compared to healthy controls (HC). **Panel b** is representing the expression of miR-148b-3p in osteoblast samples of surgical AIS cases compared to non-scoliotic trauma cases as non-scoliotic controls. GraphPad Prism 9 was used to generate graphs and statistical analysis. Statistical significance between each group was determined using the T-test for two groups and ANOVA for more than two groups (* p-value<0.05, ** p-value<0.01).

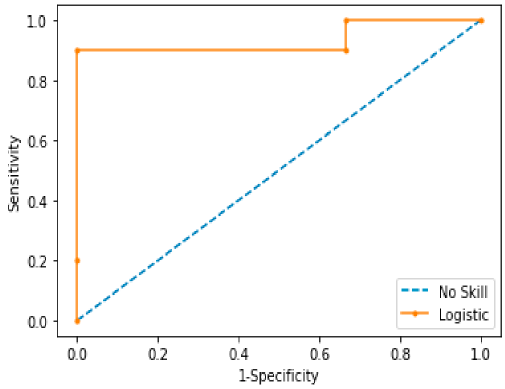

Supplementary Figure S4. Receiver-operator curve (ROC) analysis for predicting scoliosis severity in symptomatic AIS patients. ROC generated by combining the relative expression ($2^{(-\Delta\Delta CT)}$) values from five circulating miRNAs (miR-103a-3p, miR-107, miR-148a-3p, miR-148b-3p, miR-152-3p) that were upregulated in AIS progressor group compared to the non-progressor group. The combined miRNA signature showed an accuracyof 92.3%, sensitivityof 90%, specificityof 100% and an area under the curve (AUC) of 95% , indicating its potential for predicting scoliosis progression.

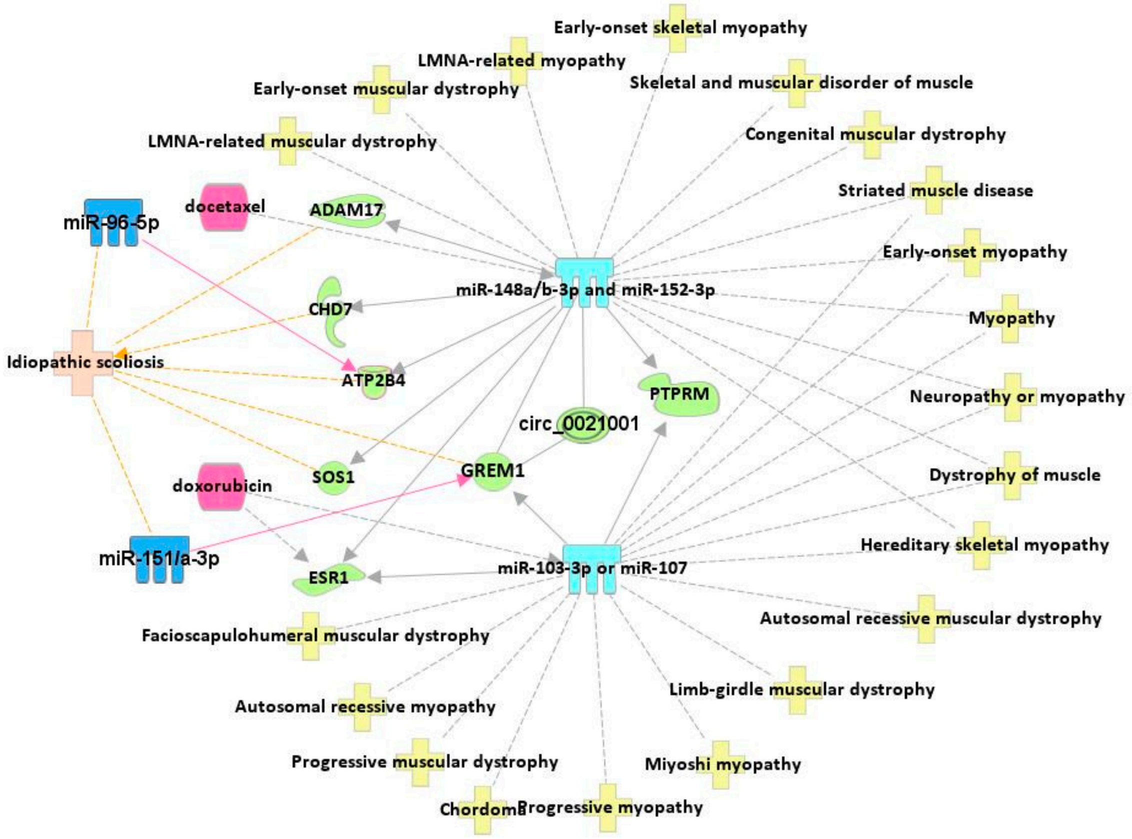

© 2000-2024 QIAGEN. All rights reserved.

Supplementary Figure S5. Five miRNAs targeting *PTPRM* gene in AIS. Using Ingenuity Pathway Analysis (IPA) software (QIAGEN Inc. software version 51,963,813) we developed a figure showing the connection of our candidate miRNAs (miR-103a-3p, miR-107, miR-148a-3p, miR-148b-3p, miR-152-3p) to the *PTPRM* gene. These miRNAs are also targeting other genes involved in idiopathic scoliosis and they play roles in different muscle and skeletal diseases. miR-151a-3p, a miRNA associated with AIS shares a common target with miR-148b-3p. The PTPRM related miRNAs are in light blue; genes targeted by our five candidate miRNAs are in light green; idiopathic scoliosis is in light pink; other diseases are represented in light yellow; drugs targeting PRPRM related genes are in pink.

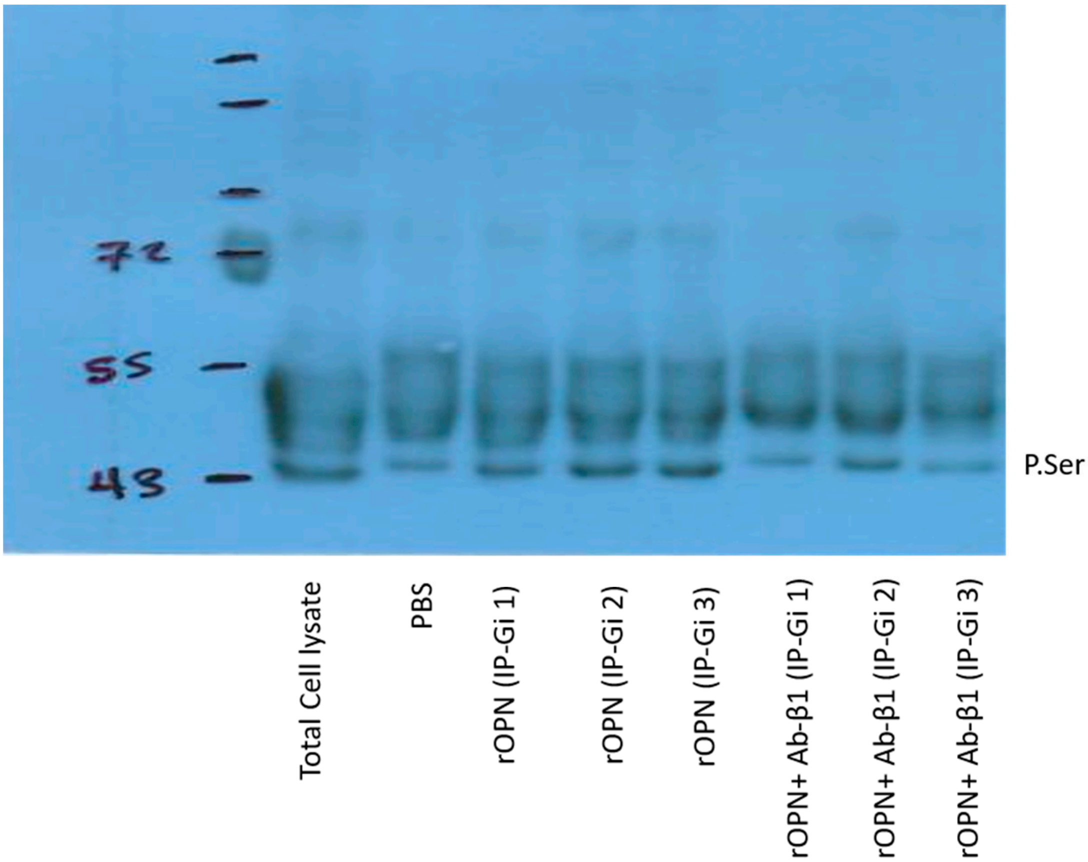

Supplementary Figure S6. Loss of PTPµ contributes to increase the affinity of α5β1 integrin toward OPN. MC3T3-E1 cells (mouse osteoblasts) treated with PBS or rOPN were subjected to immunoprecipitation with antibodies against the Gi_1, Gi_2, or Gi_3 alpha subunit. Precipitates were resolved by 10% SDS-PAGE and immunoblotted with an antibody directed against phospho-serine. Cells were pre-treated with antibody against β1 integrin for 30 min followed by an 18 h incubation with 0.5 µg/mL rOPN, prior to immunoprecipitation and immunoblotting. Bands shown are representative of results obtained with independent experiments.
